# Supplementary material for: Weekend and weekday associations between the residential built environment and physical activity: Findings from the ENABLE London study
Source: PLoS One. 2020 Sep 2;15(9):e0237323. doi: 10.1371/journal.pone.0237323 (PMC7467308; doi:10.1371/journal.pone.0237323)
Supplement: S1 Table — (DOCX) [file pone.0237323.s001.docx]

**S1 Table. Sociodemographics of ENABLE London participants included in (n=1064) and excluded from (n=214) the analyses.**

|  |  | Analytical sample | | Excluded participants | |  |
| --- | --- | --- | --- | --- | --- | --- |
|  |  | N=1064 | | N=214 | |  |
|  |  | n | (%) | n | (%) | p-value |
| Housing group | |  |  |  |  | 0.17 |
|  | Social | 222 | (22.3%) | 53 | (27.2%) |  |
|  | Intermediate | 464 | (46.6%) | 85 | (43.6%) |  |
|  | Market-rent | 310 | (31.1%) | 57 | (29.2%) |  |
| Sex | |  |  |  |  | 0.06 |
|  | Female | 621 | (58.4%) | 110 | (51.4%) |  |
| Age group | |  |  |  |  | 0.27 |
|  | 16-24 | 222 | (20.9%) | 53 | (24.8%) |  |
|  | 25-34 | 464 | (43.6%) | 85 | (39.7%) |  |
|  | 35-49 | 310 | (29.1%) | 57 | (26.6%) |  |
|  | 50+ | 68 | (6.4%) | 19 | (8.9%) |  |
| Ethnicity | |  |  |  |  | 0.18 |
|  | White | 511 | (48.0%) | 106 | (49.5%) |  |
|  | Black | 270 | (25.4%) | 53 | (24.8%) |  |
|  | Asian | 172 | (16.2%) | 42 | (19.6%) |  |
|  | Mixed/other | 111 | (10.4%) | 13 | (6.1%) |  |
