# Supplementary material for: Weekend and weekday associations between the residential built environment and physical activity: Findings from the ENABLE London study
Source: PLoS One. 2020 Sep 2;15(9):e0237323. doi: 10.1371/journal.pone.0237323 (PMC7467308; doi:10.1371/journal.pone.0237323)
Supplement: S2 Table — (DOCX) [file pone.0237323.s002.docx]

**S2 Table. Description, number and size of metropolitan, district and local parks.**

|  | **Description**  *(as in the Greater London Authority (GLA) London Plan March 2016)* | **N** | **Size (ha)**  Median [IQR] |
| --- | --- | --- | --- |
| Metropolitan parks | Large areas of open space that provide a range of facilities and features offering recreational, ecological, landscape, cultural or green infrastructure benefits, are readily accessible by public transport and are managed to meet best practice quality standards | 110 | 52.6 [73.7] |
| District parks | Large areas of open space that provide a landscape setting with a variety of natural features providing a wide range of activities, including outdoor sports facilities and playing fields, children's play areas for different age groups and informal recreation pursuits. | 145 | 20.9 [18.7] |
| Local parks | Providing for court games, children's play, sitting out areas and nature conservation areas. | 493 | 5.0 [5.8] |
