# Supplementary material for: Weekend and weekday associations between the residential built environment and physical activity: Findings from the ENABLE London study
Source: PLoS One. 2020 Sep 2;15(9):e0237323. doi: 10.1371/journal.pone.0237323 (PMC7467308; doi:10.1371/journal.pone.0237323)
Supplement: S3 Table — (DOCX) [file pone.0237323.s003.docx]

**S3 Table. Data sources and versions used for computing residential built environment variables.**

|  | **Aspirational housing group** | | |
| --- | --- | --- | --- |
|  | Social | Intermediate | Market-rent |
|  |  |  |  |
| Median date of data collection | Aug 2013 | May 2014 | May 2015 |
|  |  |  |  |
| Street network data | ITN, version June 2015 | ITN, version June 2015 | ITN, version June 2015 |
| Residential unit data | AddressBase Plus May, version 2013 | AddressBase Plus, version May 2014 | AddressBase Plus, version July 2015 |
| Topography data | OS MasterMap Topography layer, version December 2013 | OS MasterMap Topography layer May 2014 | OS MasterMap Topography layer, version June 2015 |
| Park data | GiGL, downloaded in 2015 | GiGL, downloaded in 2015 | GiGL, downloaded in 2015 |
| Public transport data | Version 2015 | Version 2015 | Version 2015 |

*Note: The median date of data collection differed between the three housing groups due to phased release of accomodation for occupancy*
