# Supplementary material for: Weekend and weekday associations between the residential built environment and physical activity: Findings from the ENABLE London study
Source: PLoS One. 2020 Sep 2;15(9):e0237323. doi: 10.1371/journal.pone.0237323 (PMC7467308; doi:10.1371/journal.pone.0237323)
Supplement: S4 Table — (DOCX) [file pone.0237323.s004.docx]

**S4 Table. Criteria for allocation of housing tenures in East Village.**

| **Housing tenure** | **Criteria for allocation** | **Provider** |
| --- | --- | --- |
| **Social** | Shortlisting (from a ‘Social Housing Register’) was based on a points system. Points were based on current living conditions, household composition (overcrowding), affordability, employment status, credit history, tenancy management, and medical circumstance (the system used to calculate these points was not available to the Investigators) | East Thames Group (association) |
| **Intermediate** | Mix of affordable market-rent, shared equity (the buyer can pay a small deposit, usually 5%, and top up to 20% of the purchase price with a low or no cost ‘equity loan’, with the remainder paid by mortgage) and shared ownership (those who are unable to obtain a mortgage on the open market). Eligibility criteria included: living or working in London; an annual household income of less than £66,000 for 1 and 2 bedroom homes or below £80,000 for 3 bedroom homes; and to be a first time buyer for shared ownership accommodation. Affordability checks were conducted before tenants were invited to view a property. | Triathlon Homes |
| **Market-rent** | Anyone seeking private rentals (usually a minimum income requirement of at least £39,000 per annum). Affordability/reference checks in place to ensure tenants could afford the accommodation | Get Living London |
