# Supplementary material for: Weekend and weekday associations between the residential built environment and physical activity: Findings from the ENABLE London study
Source: PLoS One. 2020 Sep 2;15(9):e0237323. doi: 10.1371/journal.pone.0237323 (PMC7467308; doi:10.1371/journal.pone.0237323)
Supplement: S5 Table — (DOCX) [file pone.0237323.s005.docx]

**S5 Table. Baseline demographic and daily steps of 837 ENABLE-London participants who provided daily physical activity data for weekdays and weekend days.**

|  | **Total** | | **Social** | | **Intermediate** | | **Market rent** | |  |
| --- | --- | --- | --- | --- | --- | --- | --- | --- | --- |
| **N** | 837 | | 325 | | 354 | | 158 | |  |
|  | n | (%) | n | (%) | n | (%) | n | (%) | p-value |
| **Sex:** female | 490 | (59%) | 246 | (76%) | 176 | (50%) | 68 | (43%) | <0.001 |
| **Age** |  |  |  |  |  |  |  |  | <0.001 |
| 16-24 | 161 | (19%) | 69 | (21%) | 56 | (16%) | 36 | (23%) |  |
| 25-34 | 367 | (44%) | 73 | (22%) | 208 | (59%) | 86 | (54%) |  |
| 35-49 | 248 | (30%) | 152 | (47%) | 78 | (22%) | 18 | (11%) |  |
| 50+ | 61 | (7%) | 31 | (10%) | 12 | (3%) | 18 | (11%) |  |
| **Ethnic group** |  |  |  |  |  |  |  |  | <0.001 |
| White | 414 | (49%) | 56 | (17%) | 251 | (71%) | 107 | (68%) |  |
| Black | 201 | (24%) | 155 | (48%) | 33 | (9%) | 13 | (8%) |  |
| Asian | 128 | (15%) | 65 | (20%) | 47 | (13%) | 16 | (10%) |  |
| Mixed / Other | 94 | (11%) | 49 | (15%) | 23 | (6%) | 22 | (14%) |  |
|  |  |  |  |  |  |  |  |  |  |
| **Residential Built Environment Factors** |  |  |  |  |  |  |  |  |  |
| Walkability, mean (95% CI) | -0.04 | (-0.22, 0.14) | -0.60 | (-0.82, -0.37) | 0.08 | (-0.21, 0.37) | 0.84 | (0.34, 1.35) | <0.001 |
| Distance to metropolitan parks (km), median (IQR) | 2.12 | (1.17, 3.48) | 2.56 | (1.39, 3.72) | 1.85 | (0.95, 2.99) | 1.85 | (1.04, 3.15) | <0.001 |
| Distance to district parks (km), median (IQR) | 2.19 | (1.38, 3.06) | 2.30 | (1.72, 3.25) | 2.05 | (1.04, 2.90) | 2.02 | (1.39, 2.91) | <0.001 |
| Distance to local parks (km), median (IQR) | 0.75 | (0.43, 1.18) | 0.58 | (0.36, 0.98) | 0.84 | (0.47, 1.35) | 0.88 | (0.49, 1.35) | <0.001 |
| Public transport accessibility, n (%) |  |  |  |  |  |  |  |  | <0.001 |
| Low | 78 | (9%) | 36 | (11%) | 32 | (9%) | 10 | (6%) |  |
| Intermediate | 475 | (57%) | 216 | (66%) | 180 | (51%) | 79 | (50%) |  |
| High | 284 | (34%) | 73 | (22%) | 142 | (40%) | 69 | (44%) |  |
|  |  |  |  |  |  |  |  |  |  |
| **Physical activity ^1,2^** | **mean** | **(95% CI)** | **mean** | **(95% CI)** | **mean** | **(95% CI)** | **mean** | **(95% CI)** |  |
| Daily steps on week days (n=1,053) | 9,253 | (9,028, 9,478) | 8,686 | (8,263, 9,109) | 9,702 | (9,329, 10,075) | 9,414 | (8,874, 9,954) | |
| Daily steps on weekend days (n=848) | 8,430 | (8,152, 8,708) | 6,926 | (6,405, 7,446) | 9,324 | (8,865, 9,784) | 9,523 | (8,859, 10,187) | |
| Daily minutes of MVPA on week days (n=1,053) | 61.8 | (60.1, 63.6) | 57.5 | (54.1, 60.9) | 64.3 | (61.3, 67.2) | 65.3 | (61.1, 69.6) |  |
| Daily minutes of MVPA on weekend days (n=848) | 55.4 | (53.2, 57.7) | 45.4 | (41.2, 49.6) | 60.6 | (56.9, 64.3) | 64.5 | (59.1, 69.9) |  |
|  |  |  |  |  |  |  |  |  |  |

**Footnotes**

1. Daily steps and minutes of MVPA are adjusted for sex, age group, ethnic group and housing group as fixed effects and household as a random effect in a multi-level model.

2. Differences between Social and both Intermediate and Market-rent groups were statistically significant, p<0.01.
