# Supplementary material for: Weekend and weekday associations between the residential built environment and physical activity: Findings from the ENABLE London study
Source: PLoS One. 2020 Sep 2;15(9):e0237323. doi: 10.1371/journal.pone.0237323 (PMC7467308; doi:10.1371/journal.pone.0237323)
Supplement: S6 Table — (DOCX) [file pone.0237323.s006.docx]

**S6 Table.** **Regression estimates for the association between residential built environment variables and physical activity outcomes (daily steps and daily minutes of MVPA) in the London ENABLE study for 837 participants who provided PA data on weekdays and weekend days.**

|  |  | |  | **Model 1 ^1^** | | **Model 2 ^2^** | | **Model 3 ^3^** | | **Model 1 ^1^** | | **Model 2 ^2^** | | **Model 3 ^3^** | | |
| --- | --- | --- | --- | --- | --- | --- | --- | --- | --- | --- | --- | --- | --- | --- | --- | --- |
|  |  | |  | **β** | **(95% CI)** | **β** | **(95% CI)** | **β** | **(95% CI)** | **β** | **(95% CI)** | **β** | **(95% CI)** | **β** | **(95% CI)** | |
|  |  | |  | **Outcome: daily steps on weekdays** | | | | | | **Outcome: daily steps on weekend days** | | | | | | |
|  | | Walkability | | 30 | (-58, 118) | -24 | (-111, 63) | 11 | (-90, 112) | **248** | **(132, 363)** | **126** | **(19, 233)** | **142** | **(17, 267)** |  |
|  | | Distance to closest | |  |  |  |  |  |  |  |  |  |  |  |  |  |
|  |  | | metropolitan park (km) | **-342** | **(-506, -179)** | **-246** | **(-406, -85)** | **-306** | **(-477, -135)** | **-370** | **(-589, -150)** | -127 | (-327, 72) | -147 | (-358, 64) |  |
|  |  | | district park (km) | -115 | (-302, 72) | -19 | (-201, 162) | -105 | (-297, 86) | **-346** | **(-592, -99)** | -164 | (-387, 58) | -124 | (-359, 110) |  |
|  |  | | local park (km) | 208 | (-156, 571) | -37 | (-391, 318) | -156 | (-516, 204) | **1,130** | **(654, 1,605)** | **597** | **(163, 1,031)** | **598** | **(156, 1,039)** |  |
|  | | Accessiblity to public transport | |  |  |  |  |  |  |  |  |  |  |  |  |  |
|  |  | | Low | 350 | (-523, 1,223) | 676 | (-170, 1,522) | **1,013** | **(47, 1,980)** | **-1,262** | **(-2,422, -102)** | -530 | (-1,576, 515) | 130 | (-1,063, 1,323) |  |
|  |  | | Intermediate | -443 | (-953, 67) | -126 | (-625, 373) | 11 | (-525, 548) | **-1,011** | **(-1,688, -335)** | -289 | (-906, 327) | 64 | (-599, 727) |  |
|  |  | | High (reference group) |  |  |  |  |  |  |  |  |  |  |  |  | |
|  |  | |  |  |  |  |  |  |  |  |  |  |  |  |  | |
|  |  | |  | **Outcome: daily minutes of MVPA on weekdays** | | | | | | **Outcome: daily minutes of MVPA on weekend days** | | | | | | |
|  | | Walkability | | 0.5 | (-0.2, 1.2) | 0.0 | (-0.7, 0.7) | 0.2 | (-0.6, 1.1) | **2.1** | **(1.2, 3.1)** | **1.2** | **(0.3, 2.0)** | **1.4** | **(0.4, 2.5)** | |
|  | | Distance to closest | |  |  |  |  |  |  |  |  |  |  |  |  | |
|  |  | | metropolitan park (km) | **-2.8** | **(-4.1, -1.5)** | **-1.9** | **(-3.2, -0.6)** | **-2.3** | **(-3.6, -0.9)** | **-2.9** | **(-4.7, -1.2)** | -1.1 | (-2.7, 0.5) | -1.2 | (-2.9, 0.5) | |
|  |  | | district park (km) | -0.7 | (-2.2, 0.8) | 0.2 | (-1.3, 1.6) | -0.4 | (-1.9, 1.1) | **-2.3** | **(-4.3, -0.3)** | -0.9 | (-2.7, 0.9) | -0.4 | (-2.3, 1.5) | |
|  |  | | local park (km) | 1.9 | (-1.0, 4.9) | -0.3 | (-3.1, 2.5) | -1.0 | (-3.9, 1.8) | **9.0** | **(5.1, 12.8)** | **4.8** | **(1.2, 8.3)** | **4.9** | **(1.4, 8.5)** | |
|  | | Accessiblity to public transport | |  |  |  |  |  |  |  |  |  |  |  |  | |
|  |  | | Low | 1.4 | (-5.7, 8.5) | 4.3 | (-2.4, 11.0) | 7.5 | (-0.2, 15.2) | -9.1 | (-18.5, 0.3) | -3.7 | (-12.2, 4.7) | 2.9 | (-6.8, 12.5) | |
|  |  | | Intermediate | **-4.2** | **(-8.3, -0.1)** | -1.5 | (-5.5, 2.4) | -0.2 | (-4.5, 4.0) | **-7.7** | **(-13.1, -2.2)** | -2.5 | (-7.5, 2.5) | 0.9 | (-4.5, 6.2) | |
|  |  | | High (reference group) |  |  |  |  |  |  |  |  |  |  |  |  | |
|  |  | |  |  |  |  |  |  |  |  |  |  |  |  |  | |

**Footnotes**

Total number=1064, data collected 2013-2016. Effect estimates highlighted in **bold** are statistically significant, p<0.05.

1. Model 1 adjusts for household as a random effect to allow for clustering at the household level (referred to as “minimally adjusted model” in the text).

2. Model 2 additionally adjusts for sex, age group, ethnic group, aspirational housing group as fixed effects.

3. Model 3 additionally adjusts for all residential built environment variables as fixed effects.
