# Supplementary material for: Weekend and weekday associations between the residential built environment and physical activity: Findings from the ENABLE London study
Source: PLoS One. 2020 Sep 2;15(9):e0237323. doi: 10.1371/journal.pone.0237323 (PMC7467308; doi:10.1371/journal.pone.0237323)
Supplement: S7 Table — (DOCX) [file pone.0237323.s007.docx]

**S7 Table.** **Effect of adjustment for residential built environment factors in the differences in daily physical activity by housing group in the London-ENABLE study in 837 participants who provided PA data on both weekdays and weekend days.**

|  |  |  | | **Base model with further adjustment for** | | | | | | | | | | | |
| --- | --- | --- | --- | --- | --- | --- | --- | --- | --- | --- | --- | --- | --- | --- | --- |
|  |  | **Base model ^1^** | | **Walkability** | | **Distance to metropolitan park** | | **Distance to**  **district park** | | **Distance to**  **local park** | | **Accessibility to**  **public transport** | | **All BE variables** | |
|  | **Housing group** | **β** | **(95% CI)** | **β** | **(95% CI)** | **β** | **(95% CI)** | **β** | **(95% CI)** | **β** | **(95% CI)** | **β** | **(95% CI)** | **β** | **(95% CI)** |
| **Daily steps** |  |  |  |  |  |  |  |  |  |  |  |  |  |  |  |
| Weekday, n=1053 | Social (reference group) | | |  |  |  |  |  |  |  |  |  |  |  |  |
|  | Intermediate | 1,016 | (401, 1,632) | 1,022 | (406, 1,638) | 930 | (316, 1,545) | 1,012 | (395, 1,629) | 1,023 | (404, 1,642) | 1,004 | (384, 1,624) | 922 | (300, 1,544) |
|  | Market-rent | 729 | (0, 1,458) | 756 | (20, 1,492) | 653 | (-73, 1,380) | 726 | (-5, 1,456) | 736 | (3, 1,468) | 741 | (5, 1,476) | 682 | (-56, 1,420) |
|  |  |  |  |  |  |  |  |  |  |  |  |  |  |  |  |
| Weekend n=848 | Social (reference group) | | |  |  |  |  |  |  |  |  |  |  |  |  |
|  | Intermediate | 1,016 | (401, 1,632) | 1,022 | (406, 1,638) | 930 | (316, 1,545) | 1,012 | (395, 1,629) | 1,023 | (404, 1,642) | 1,004 | (384, 1,624) | 922 | (300, 1,544) |
|  | Market-rent | 729 | (0, 1,458) | 756 | (20, 1,492) | 653 | (-73, 1,380) | 726 | (-5, 1,456) | 736 | (3, 1,468) | 741 | (5, 1,476) | 682 | (-56, 1,420) |
|  |  |  |  |  |  |  |  |  |  |  |  |  |  |  |  |
| **Daily minutes of MVPA** | |  |  |  |  |  |  |  |  |  |  |  |  |  |  |
| Weekday n=1053 | Social (reference group) | | |  |  |  |  |  |  |  |  |  |  |  |  |
|  | Intermediate | 6.8 | (1.9, 11.6) | 6.8 | (1.9, 11.7) | 6.1 | (1.2, 11.0) | 6.8 | (1.9, 11.7) | 6.8 | (1.9, 11.7) | 6.6 | (1.7, 11.5) | 6.0 | (1.1, 11.0) |
|  | Market-rent | 7.8 | (2.1, 13.6) | 7.9 | (2.1, 13.7) | 7.2 | (1.5, 13.0) | 7.9 | (2.1, 13.6) | 7.9 | (2.1, 13.7) | 7.8 | (2.0, 13.6) | 7.3 | (1.4, 13.1) |
|  |  |  |  |  |  |  |  |  |  |  |  |  |  |  |  |
| Weekend n=848 | Social (reference group) | | |  |  |  |  |  |  |  |  |  |  |  |  |
|  | Intermediate | 15.2 | (9.1, 21.3) | 14.9 | (8.8, 21.0) | 14.9 | (8.7, 21.0) | 15.0 | (8.9, 21.1) | 14.4 | (8.2, 20.5) | 14.8 | (8.6, 21.0) | 13.6 | (7.4, 19.8) |
|  | Market-rent | 19.1 | (11.9, 26.4) | 17.7 | (10.5, 25.0) | 18.8 | (11.5, 26.0) | 19.0 | (11.7, 26.2) | 18.2 | (11.0, 25.5) | 18.6 | (11.3, 25.9) | 16.3 | (9.0, 23.7) |
|  |  |  |  |  |  |  |  |  |  |  |  |  |  |  |  |

**Footnotes**

1. The base model is adjusted for sex, age group, ethnic group as fixed effects and household as a random effect to allow for clustering in a multi-level model.
