# Supplementary material for: Weekend and weekday associations between the residential built environment and physical activity: Findings from the ENABLE London study
Source: PLoS One. 2020 Sep 2;15(9):e0237323. doi: 10.1371/journal.pone.0237323 (PMC7467308; doi:10.1371/journal.pone.0237323)
Supplement: S1 Fig — Legend: Mean (95%CI) daily steps and mean (95%CI) daily MVPA (min) on weekdays (n = 1,053) and on weekend days (n = 848) plotted against A) walkability scores in the residential area at each quintile (median value), B) distance to the closest local park at each quintile (median value), C) distance to the closest district park at each quintile (median value), D) distance to the closest metropolitan park at each quintile (median value), E) accessibility to public transport. Means are adjusted for sex, age group, ethnic group, aspirational housing tenure and clustering at household level. (DOCX) [file pone.0237323.s008.docx]

**S1 Fig. Daily steps and MVPA on weekends and on weekdays plotted against quintile of residential built environmental factors.**

A)

B)

C)

D)

E)

Legend: Mean (95%CI) daily steps and mean (95%CI) daily MVPA (min) on weekdays (n=1,053) and on weekend days (n=848) plotted against A) walkability scores in the residential area at each quintile (median value), B) distance to the closest local park at each quintile (median value), C) distance to the closest district park at each quintile (median value), D) distance to the closest metropolitan park at each quintile (median value), E) accessibility to public transport. Means are adjusted for sex, age group, ethnic group, aspirational housing tenure and clustering at household level
